# Social Support and Living Situation of Older Adults With Hip-Fracture: A Retrospective Cohort Study

**DOI:** 10.1093/geroni/igab046.2352

**Published:** 2021-12-17

**Authors:** Alexandra Krassikova, Steven Stewart, Jennifer Bethell, Aileen Davis, Katherine McGilton

**Affiliations:** 1 University of Toronto, Univerity of Toronto, Ontario, Canada; 2 Toronto Rehabilitation Institute, Toronto Rehabilitation Institute, Ontario, Canada; 3 Toronto Rehabiliitation Institute, Toronto Rehabilitation Institute, Ontario, Canada; 4 University of Toronto, University of Toronto, Ontario, Canada; 5 KITE-Toronto Rehabilitation, University Health Network, Toronto, Ontario, Canada

## Abstract

Sustaining a hip-fracture is a life-changing event negatively affecting older adults. Although, social support is a known determinant of health outcomes, the relationship between social support and living situation of older adults with hip fracture remains under researched. For this study social support is conceptualized using the Finfgeld-Connett framework, where social support is seen as being composed of emotional and instrumental support. The objectives were to examine the relationship between two domains of social support and living situation: 1) after discharge; 2) 3-months after discharge; and 3) 6-months after discharge from an inpatient rehabilitation facility in a sample of older adults with hip fracture. Emotional support was measured as frequency of interaction with someone one week prior to hip fracture, whereas instrumental support was measured as help received in instrumental activities of daily living. Logistic regression was performed to examine the association between social support and living situation. Majority of study participants (N=139) were older (mean age 81.31), female (77.70%), had no cognitive impairment (68.35%), were not married (58.99%), and lived with someone (51.80%) in their own house (71.95%). Older adults with more emotional support were more likely to be discharged home, however little can be said about the effect of the association (OR 6.80, 95% CI 1.08, 22.31, P<.001). Persons receiving more instrumental support had less odds of living at home 3-months (OR 0.41, 95% CI 0.21, 0.78; P=.007) and 6-months after discharge (OR 0.59, 95% CI 0.38, 0.91, P=0.017). Social support is important for older adults during recovery.

